# The Potential Role of Chemerin, Lipocalin 2, and Apelin in the Diagnosis and Pathophysiology of Gestational Diabetes Mellitus

**DOI:** 10.1155/2021/5547228

**Published:** 2021-06-09

**Authors:** Radzisław Mierzyński, Elżbieta Poniedziałek-Czajkowska, Dominik Dłuski, Maciej Kamiński, Agnieszka Mierzyńska, Bożena Leszczyńska-Gorzelak

**Affiliations:** Chair and Department of Obstetrics and Perinatology, Medical University of Lublin, 20-954 Lublin, Poland

## Abstract

The exact role of adipokines in the pathogenesis of gestational diabetes mellitus (GDM) still remains not fully clear, and multiple studies have analyzed their potential contribution to the pathophysiology of this pregnancy complication. This study is aimed at evaluating serum chemerin, lipocalin 2, and apelin concentrations in GDM and healthy pregnant patients, assessing the correlation between these adipokines, and suggesting the potential role of these cytokines in the diagnosis and pathophysiology of GDM. The study comprised 237 pregnant women: 153 with GDM and 84 with physiological pregnancy. Serum concentrations of chemerin, lipocalin 2, and apelin were obtained at 24–29 weeks of gestation. The mean concentrations of chemerin and lipocalin 2 were significantly higher in the GDM group. The concentration of apelin was slightly higher in the GDM group, but not statistically significant. The strong positive correlation between chemerin and lipocalin 2 concentrations was noticed in both groups. Our data suggest that maternal chemerin and lipocalin 2 may play a significant role in the pathophysiology of GDM. We imply that these adipokines could potentially be established as novel biomarkers for the early identification of GDM. However, more studies are needed to analyze the effect of these adipokines on glucose metabolism during early pregnancy.

## 1. Introduction

Gestational diabetes mellitus (GDM) is the most frequent medical and metabolic complication characterising pregnant women. GDM affects from 5 to 20% of all pregnancies, depending on the ethnicity, screening method employed, and the diagnostic tests used [1]. GDM is associated with a higher risk of fetal a maternal adverse outcomes (macrosomia, hypertensive disorders, cesarean section, asymmetrical intrauterine growth retardation, stillbirth, neonatal, hyperbilirubinemia, hypoglycemia, hypocalcemia, polycythemia, and neonatal respiratory distress) [2, 3]. It should be emphasized that GDM patients have also a significantly increased risk for the development of type 2 diabetes mellitus (T2DM) and cardiovascular morbidity and mortality in future life [4]. Their offspring are at higher risk of fostering obesity and impaired glucose metabolism in later life. During pregnancy, an adaptation of maternal metabolism with increased nutritional requirements to support growth is observed [4]. Pregnancy is also characterized by decreased insulin sensitivity [5]. Decreased maternal prepregnancy insulin sensitivity and preconception insulin resistance, impaired insulin response during the pregnancy, and insulin-producing *β*-cells dysfunction are believed to be the most important components of the pathophysiology of GDM development [5]. However, insulin resistance is considered a physiological metabolic change during pregnancy, which provides a suitable concentration of glucose for the metabolic needs of the rapidly growing fetus.

Although our research was performed before the COVID-19 pandemic outbreak, at present, we have to remember the influence of this situation on the prevalence of GDM [6, 7]. The pandemic lockdown because of a decrease in physical activity and modifications in patients' dietary habits, increased consumption of snacks, unhealthy foods, and sweets, may influence body weight. The metabolic changes include an increase in insulin resistance, total body fat, abdominal fat, and inflammatory cytokines. These factors have been shown to correlate with the higher risk of GDM. It is suggested that the Mediterranean diet could be considered, especially during a pandemic, as a useful dietary option during pregnancy to decrease the risk of maternal-fetal complications [7]. Another possible mechanism for the increased number of women with GDM may be the greater anxiety associated with the COVID-19 lockdown. The stress that pregnant women have experienced during the lockdown could initiate a cascade of endocrinological and immunological alterations that affect the delicate equilibrium necessary to maintain a physiological pregnancy and can cause the development of pregnancy complications. It is suggested that excessive activity of circulating cortisol may increase insulin resistance, a typical feature in the pathogenesis of GDM [6].

Numerous metabolic changes observed during the pregnancy appear to be influenced by adipokines [8]. It has been described that adipokines may play a key role in maternal-fetal metabolic adaptations and are involved in numerous metabolic processes. They modulate placental function and may have a significant impact on fetal development. Abnormal production or secretion of adipokines is observed in insulin resistance [8]. The significance of adipokines in the pathogenesis of GDM is still not well known. The dysregulation of several adipokines metabolism and/or placental function may play a crucial role in the pathophysiology of GDM [9].

Different adipokines have been analyzed as biomarkers for GDM; however, no marker has been reported for GDM screening so far [5].

Chemerin is a novel chemoattractant 14 kDa protein, described as retinoic acid receptor responder protein 2 (RARRES2), secreted as a prochemerin. This inactive precursor is changed into the active molecule by coagulation and inflammatory serine proteases [10]. Chemerin and the receptor of chemerin, chemokine-like receptor 1 (CMKLR1, also known as ChemR23) are almost exclusively expressed and synthesized in white adipose tissue [11]. Swensson et al. confirm that adipokines such as chemerin are also produced in several tissues apart from adipose tissue including human serum albumin [12].

Chemerin plays an important role in adipocyte differentiation, and insulin signaling results in an impact on the regulation of inflammation and major metabolic processes [10]. Its elevated levels are observed in obesity and metabolic syndrome [10]. The increased level of chemerin that occurs with obesity is hypothesized to play a substantial role in the development of T2DM as a result of dysregulation of the essential pathophysiological processes modified by chemerin [10]. It has been also described that chemerin might be an independent predictor of T2DM and cardiovascular events [13]. Recent studies have also postulated that chemerin may play an essential role in the pathophysiology of GDM [11]. Some authors notice that markedly increased circulating chemerin levels in peripheral blood are observed in GDM patients [14, 15]. It has been also suggested in the first and second trimester of pregnancy logistic multivariate regression analysis that chemerin concentrations are positively correlated with the increased risk of GDM, and together with other factors, chemerin can be used as an independent risk factor of gestational diabetes mellitus [14, 15].

Lipocalins are a superfamily of proteins characterized by a range of different molecular-recognition features [16]. Lipocalin 2 (LCN2), also known as neutrophil gelatinase-associated lipocalin (NGAL), was first found in human neutrophils and is also expressed in adipose tissue, liver, and kidneys [17]. Numerous inflammatory stimuli, such as lipopolysaccharides and interleukin-1, can significantly induce lipocalin-2 expression and secretion [18]. LCN2 plays a crucial role in the protection of matrix metallopeptidase 9 (MMP-9) from degradation and is upregulated in pathological situations as well as cancer [18, 19]. LCN2 is one of the transcripts, which are expressed in the pregnant myometrium [20]. It is suggested that LCN2 is a possible mediator that joins obesity with chronic low-grade inflammation [21]. LCN2 has also been proved to be an inflammatory marker closely associated with insulin resistance and hyperglycemia [21].

Apelin is the natural ligand of the orphan G-protein coupled APJ receptor [22]. Apelin is produced as prepropeptide consisting of 77 amino acids and shorter biologically active forms with 12, 13, 16, 17, and 36 amino acids. The most active biological fragment is probably apelin-13. Apelin acts at peripheral tissues and the central nervous system, where it takes part in glucose metabolism [23], immune system responses, inotropy, brain signaling pathway, hemodynamic homeostasis, angiogenesis, vasodilation [24], and oxidative stress-linked atherosclerosis [25]. The presence of apelin and its receptor has also been identified in adipose tissue, where their production is regulated by nutritional status. Its expression is decreased by fasting and upregulated by refeeding [26]. The increased levels of apelin are observed in obesity-associated hyperinsulinemia [23]. Animal studies have revealed that apelin can improve glucose metabolism; therefore, it has been suggested that apelin could be a promising therapeutic target in the treatment of insulin resistance [26]. The presence of apelin has been described in human placental tissue, suggesting a crucial role of this peptide in pregnancy [27].

The role of chemerin, lipocalin 2, and apelin in the pathogenesis of GDM still remains not fully clear, and the relationship between circulating concentrations of these adipokines and risk of GDM is not well known.

We aimed to investigate serum chemerin, lipocalin 2, and apelin levels in patients diagnosed with gestational diabetes and healthy pregnant patients, to analyze the relationship between these adipocytokines, and to discuss the potential role of these cytokines in the diagnosis and pathophysiology of GDM.

## 2. Materials and Methods

The prospective study was conducted on 153 pregnant patients with diagnosis of gestational diabetes and 84 patients with uncomplicated pregnancy and was performed in the Chair and Department of Obstetrics and Perinatology, Medical University of Lublin, Poland. Patients signed informed decision about participation in the clinical investigation. Approval for the trial was obtained from the Bioethical Review Board of the Medical University of Lublin (No. KE-0254/117/2018). The research was conducted in accordance with the principles published in the Declaration of Helsinki.

Inclusion criteria for the study group were the following: gestational age between 24 and 29 weeks, first prenatal visit before 10 weeks of gestation, singleton pregnancy, and gestational diabetes first recognized in the present pregnancy before 28 weeks of gestation.

**Figure 1:**
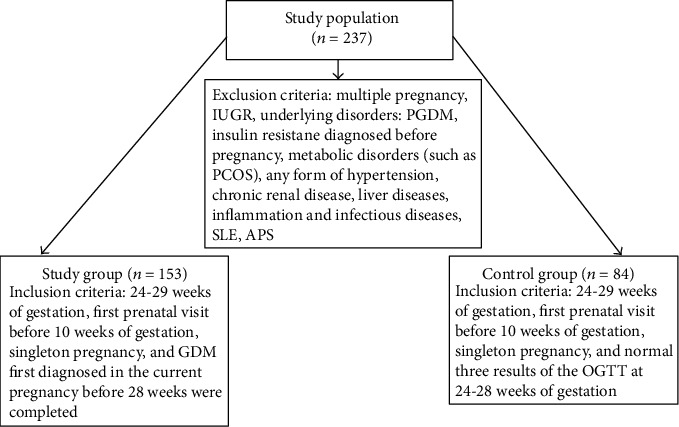
Flow chart of study population.

Inclusion criteria for the control group were the following: gestational age between 24 and 29 weeks, first prenatal visit before 10 weeks of gestation, singleton pregnancy, and normal three results of the oral glucose tolerance test (OGTT) at 24–28 weeks of gestation (Figure 1).

Those patients with multiple pregnancy, intrauterine growth restriction (IUGR), concomitant disturbances: pregestational diabetes mellitus (PGDM), insulin resistance diagnosed before pregnancy, metabolic disorders (such as polycystic ovary syndrome—PCOS), hypertensive disorders, chronic renal and liver diseases, inflammatory and infectious diseases, systemic lupus erythematosus (SLE), and antiphospholipid syndrome (APS) were excluded from the study.

All participants had undergone screening for GDM with a 75 g OGTT at 24–28 weeks' gestation, according to WHO standards. GDM was diagnosed if at least one of the threshold values was met: fasting glucose level 5.1–6.9 mmol/L (92–125 mg/dL) at 1st hour ≥10.0 mmol/l (180 mg/dL) and at 2nd hour 8.5–11.0 mmol/L (153–199 mg/dL) [28].

Data on present pregnancy and history of previous pregnancies, maternal and family history, maternal age, and infant outcome were received by analyzing medical records.

Prepregnancy body mass index (BMI) was computed as reported weight prior to pregnancy (kg) divided by square of measured height (m). Height was measured at baseline by trained research assistants, with a wall-mounted stadiometer and shoes taken off. Weight was measured on a digital scale with 100 g resolution and capacity of 150 kg. The participants were wearing light clothing and no shoes. BMI was recalculated when the blood samples were taken.

The blood specimens for research analysis were taken at the same time when the blood specimens have been taken for routinely performed laboratory analysis. Serum levels of chemerin, lipocalin, and apelin were analyzed at 24–29 weeks of pregnancy. The samples were allowed to sit for at least 30 minutes and then centrifuged at 2000 gravitational units (g) for 20 minutes. Afterwards, serum was removed and then stored at −70°C. The chemerin level assay was performed with ELISA kit (Human Chemerin, BioVendor R&D Products, Czech Republic), as well as the lipocalin level (Human Lipocalin-2/NGAL, BioVendor R&D Products, Czech Republic), and apelin concentration (Human Apelin, Cloud-Clone Corp., USA). The limit of chemerin detection was 0.1 ng/ml. The intra- and interassay coefficients of variation (CVs) were 5.1% and 8.6%, respectively. The limit of lipocalin detection was 0.02 ng/mL, while the intra-assay and interassay coefficients of variation were 7.0% and 9.8%, respectively. The limit of apelin detection was 8.25 pg/ml. The intra- and interassay coefficients of variation (CVs) were <10% and <12%, respectively.

The patient's age, gravidity, gestational age at baseline, pregestational BMI, BMI at blood collection, estimated fetal weight (EFW) at sampling, and OGTT hourly glucose levels, as well as chemerin, lipocalin 2, and apelin levels were investigated. Correlations between chemerin, lipocalin, apelin and BMI, maternal age, gravidity, EFW, and OGTT hourly glucose levels were analyzed.

All statistical analyses were performed using STATISTICA, v. 12.0 (StatSoft, Inc., Tulsa, OK, USA). For variables of normal distribution and homogenous variances, difference significances were determined using a one-way analysis of variance (ANOVA) followed by Tukey's *post hoc* test. The Shapiro-Wilk test for normal distribution of data and one-tailed Student's *t*-test, or (in unequal variance) the Cochran-Cox test (absence of normal distribution and non-parametric data), and the Mann–Whitney *U* test, were all done. Results with normal distribution were presented as the means ± standard deviation (SD). The correlation analysis was conducted using Pearson's and Spearman's correlation tests. Significance was set at *p* < 0.05. Univariate and multivariate logistic regression analyses were performed for calculations odds ratios (ORs) with 95% confidence intervals (CIs) predicting gestational diabetes mellitus based on chemerin and lipocalin serum levels. The diagnostic value of the dependent variables—chemerin and lipocalin serum level, as the predictors of the GDM, was assessed using multiple linear regression analysis. The model was performed including independent variables such as BMI before and during the pregnancy, maternal age, gestational age, and the estimated fetal weight at the day of the sample collection. As the serum level of the apelin has not differed significantly between the GDM and healthy patients groups, it was not included in the univariate and multivariate logistic regression models.

## 3. Results

There were no significant differences between the GDM group and the control group with regard to maternal age, gravidity, EFW, gestational age, and BMI at blood collection. Pregestational BMI was significantly higher in GDM patients as compared with uncomplicated pregnancy group (23.71 ± 2.64 vs. 22.81 ± 2.05 kg/m^2^, *p* < 0.05) (Table 1). The highest prepregnancy BMI value was 27.2 kg/m^2^ in the GDM group and 24.6 kg/m^2^ in the control group.

In oral fasting glucose tolerance test, at 1st and 2nd hour of test, the glucose concentrations were markedly higher in the GDM group than in the control group (Table 2).

**Figure 2:**
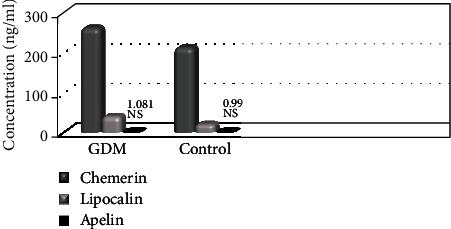
Chemerin, lipocalin 2, and apelin in GDM and control groups.

The mean chemerin concentration was significantly higher in the GDM group than in the control group (259.55 ± 63.24 vs. 211.00 ± 49.38 ng/mL, *p* < 0.0001). The mean lipocalin 2 concentration was also significantly higher in the GDM group as compared with the control group (40.49 ± 15.73 vs. 20.63 ± 7.48 ng/mL, *p* < 0.0001). The concentration of apelin was slightly higher in the GDM patients but the difference was not statistically significant (10816.45 ± 7329.52 vs. 9988.24 ± 5056.90, *p* = 0.71) (Table 2, Figure 2).

The strong positive correlation between chemerin and lipocalin 2 levels was observed in the GDM group (*R* = 0.631, *p* < 0.0001) and in the control group (*R* = 0.635, *p* < 0.0001) (Table 3). There was no correlation between chemerin and apelin levels and lipocalin 2 and apelin levels.

The correlations between chemerin, lipocalin 2 and apelin levels, and demographic and clinical features (patient's age; gravidity; pregestational BMI and BMI at blood collection; weeks of gestation and EFW at blood collection; OGTT hourly glucose concentrations) were evaluated for the GDM group and control group.

Chemerin level was positively associated with pregestational BMI, and BMI at blood collection in the GDM patient group (*R* = 0.775, 0.693, respectively), and in the control one (*R* = 0.500, 0.493, respectively) (Table 3).

There was a significant positive correlation between lipocalin 2 levels and pregestational, and at blood collection BMI in GDM patient group (*R* = 0.467 and 0.394, respectively), and in the control one (*R* = 0.311, 0.276, respectively) (Table 3). No correlation between apelin and BMI was observed.

A statistically significant relationship between chemerin levels and all values of OGTT hourly glucose concentrations were noticed in GDM patients (*R* = 0.528, 0.731, and 0.503, respectively) and in the control group (*R* = 0.817, 0.740, and 0.707, respectively). A correlation between lipocalin 2 levels and OGTT hourly glucose levels was also observed in the GDM group: *R* = 0.266, 0.425, and 0.491, respectively, and in the control one: *R* = 0.553, 0.511, and 0.423, respectively. No correlations between apelin levels and OGTT values were observed. Lipocalin 2 levels were also correlated with maternal age in the GDM group and gravidity in the control group.

The univariate linear regression model which was performed for chemerin and lipocalin has shown that the growth of each substance serum level similarly increases the likelihood of the GDM incidence in the analyzed group of patients—18% for each 10 ng/ml of chemerin and 20% for each 1 ng/ml of lipocalin (CI 95%, OR: 1,180 vs. 0.200, respectively).

In the multiple linear regression analysis of the patients with gestational diabetes, we have established that the adjusted *R*-square for chemerin was significantly elevated as compared to lipocalin (46.10 vs. 20.60, respectively).

## 4. Discussion

Our data demonstrate that pregnant women with GDM are characterized by a significantly higher concentration of chemerin and LCN2 and not significantly higher level of apelin. However, the role of these adipokines as pro- or anti-inflammatory factors is controversial.

One of the disadvantages of our study is that we did not analyze the cord blood or placenta tissue for adipokines which would have also been useful in coming to a better understanding of GDM. The clinical utility of our findings has remained limited due to the relatively small number of patients. Thus, the analysis of outcomes may be underpowered.

Another disadvantage is that maternal obesity may also influence the expression of several adipokines in the adipose tissue and the placenta. In our study, the mean prepregnancy BMI was 23.71 in the GDM group and 22.81 in the control one and the next studies should be conducted in patients with higher BMI.

Numerous adipokines have been studied during pregnancy, and their concentrations have been suggested as biomarkers of pregnancy complications, some of them with pathophysiological signification. There are controversies in the literature about the concentrations of different adipokines and their role during pregnancy. The discrepancies may be caused by the time of maternal blood sampling, laboratory methods used for analysis, sample size, and population differences.

In our study, we focused on adipokines, which are dysregulated in GDM, and three of them have been analyzed: chemerin, lipocalin 2, and apelin. To the best of our knowledge, this is the first study to compare circulating levels of chemerin, lipocalin 2, and apelin in the same group of women with gestational diabetes and those with a normal pregnancy. We also evaluated the serum levels of these adipokines in GDM and uncomplicated pregnancy patients in comparison to clinical and demographic parameters.

We noticed that the mean chemerin level was significantly higher in the GDM group than in the control one. Our results are found to be compatible with a previously published studies [29–33]. Additionally, in the study presented by Li et al., the concentrations of chemerin in all GDM groups were increased in comparison to the normal-weight-NGT group, but the chemerin level in the obese-GDM group was significantly lower than in the normal-weight-GDM and overweight-GDM group [30]. The significantly higher levels of chemerin in the third trimester in comparison to the first trimester of pregnancy were also revealed [29, 31, 34]. It is postulated that it can be associated with proinflammatory conditions because of increased levels profile of mediators of inflammation such as TNF-*α*, resistin, or IL-6 [34]. Interestingly, Yang and colleagues reported that the level of chemerin in the third trimester in the GDM group was markedly higher than in the NGT group, but the serum concentration of chemerin in the first trimester was lower in the GDM group than in the NGT group. The limitation of the study was small groups: 19 patients with GDM and 20 NGT women [31]. We also found that chemerin levels were correlated with pregestational BMI, BMI at sampling in the GDM group, and in the control group. Kasher-Meron et al. presented results, which are in line with our findings [29]. In the study conducted by Ademoglu et al., in multiple linear regression analyses they noticed that chemerin level was markedly correlated not only with BMI but also with HDL-cholesterol, triglyceride, HbA1c, insulin concentrations, and homeostasis model assessment of insulin resistance (HOMA-IR) [32]. Interestingly, fasting insulin level was comparable in both groups.

However, the HOMA-IR tended to be higher in patients with GDM but did not reach statistical significance. In the presented study, we were not able to obtain the data on the insulin concentration of all patients, but in the smaller groups (55 GDM patients and 23 controls), no correlations between the chemerin and HOMA-IR were confirmed.

In our study, in both groups, there were no obese patients with prepregnancy and at sampling BMI > 30 kg/m^2^. It is important that the highest prepregnancy BMI value was 27.2 kg/m^2^ in the GDM group and 24.6 kg/m^2^ in the control group. We excluded from our study the patients with prepregnancy diabetes mellitus, insulin resistance diagnosed before pregnancy, metabolic disorders (such as polycystic ovary syndrome), and any form of hypertension. So, the risk of markedly higher insulin resistance in our study group as compared to the control group was relatively small. Our study results showed a statistically significant correlation of chemerin levels and the OGTT hourly glucose levels in both groups. These observations are partially in line with the study presented by Fatima et al. [33]. In this analysis, chemerin level was positively associated with fasting glucose level, and additionally with HOMA-IR, and EFW.

The chemerin concentrations of both venous and arterial umbilical cord blood in newborns were also sampled and analyzed. Increased chemerin level in arterial cord blood in GDM group as compared that in control one was found but the concentrations in venous cord blood were comparable in both groups. Chemerin concentration in venous cord blood was increased in newborns of obese patients. Arterial and venous chemerin values were correlated with maternal chemerin values at the time of delivery. It has been noticed that chemerin value in arterial blood was associated with gestational diabetes status [35]. The opposite observations have been described by Barker and colleagues. In this study, no effect of GDM on maternal and cord chemerin levels was noticed as well as no change in the release of chemerin from the placenta and adipose tissue [36]. Because in our study, we did not analyze the chemerin values in arterial and venous umbilical cord blood, and we cannot compare our findings with these observations.

In a meta-analysis performed by Zhou et al., they revealed that the higher levels of circulating chemerin were correlated with GDM, and, according to the authors, this suggests that chemerin might play an essential role in the pathophysiology of GDM. They noticed that the increased chemerin levels were found in the second trimester of pregnancy as compared to women in the third trimester of pregnancy. This could be explained by the fact that serum albumin concentrations usually decrease during late pregnancy, and chemerin is released from human serum albumin. However, according to Zhou et al., these results should be interpreted with caution due to essential heterogeneity between studies, and further prospective cohort studies are needed to determine these observations [37].

Despite the growing evidence supporting a link between chemerin and GDM, the details of the mechanisms involved are unknown. The altered chemerin concentrations in GDM patients may cause insulin resistance, and an increased concentration in physiological pregnancy may have a protective role to decrease pregnancy-related insulin resistance [28]. Chemerin influences on the production of proinflammatory cytokines, chemokines, and matrix metalloproteinases (MMPs) [35, 38]. It has been also described that the administration of chemerin reduces glucose tolerance, decreases serum insulin levels, and lowers basal glucose uptake in diabetic mice *in vivo* [31]. As a result, the abnormalities in chemerin concentrations may be correlated with the development of GDM through lower insulin sensitivity and impaired anti-inflammatory capacity.

Increased concentrations of lipocalin 2 were described in metabolic diseases such as T2DM, preeclampsia, and PCOS [39, 40]. There have been published several studies describing pregnancy-related LCN2. However, we have found very few articles analyzing LCN2 in relation to GDM [41–44].

In the presented study, we have found higher LCN2 concentrations in the GDM group than in the control one. Similar observations have been published by Edelstam and colleagues, who noticed that LCN2 levels were elevated during the third trimester of pregnancy and additionally significantly increased postpartum [43].

In the study published by Lou et al., LCN2 concentration in GDM overweight and nonoverweight women were markedly higher in comparison to NGT women. LCN2 level was also markedly higher in GDM overweight than in GDM nonoverweight group. There were also positive correlations between LCN2 and parameters of insulin resistance: fasting plasma glucose (FGP), HOMA-IR, fasting plasma insulin (FPI), high-sensitivity C-reactive protein (hs-CRP), total cholesterol, and triglyceride. Furthermore, the expression of LCN2 mRNA and protein in subcutaneous adipose tissue (SAT) was higher in obese women. The researchers suggested that LCN2 could act in the development of insulin resistance in GDM, and its expression in subcutaneous adipose tissue may be associated with obesity in GDM women [42]. These results coincide with our outcomes. We found a significant correlation between LCN2 levels and pregestational BMI, and BMI at sampling, and additionally with OGTT glucose levels in both groups. However, as in chemerin results, we were not able to obtain the data on the insulin concentration of all patients, but in the smaller groups (55 GDM patients and 23 controls), no correlations between the LCN2 and HOMA-IR were revealed.

Additionally, in our study, in the GDM group, the weak positive correlation between LCN2 levels and maternal age at the blood collection has been noticed. The relationship between age and adipokine levels is ambiguous [44]. There appear to be no data in the literature on the possible mechanisms of such correlation and clinical implications, and we cannot compare our findings with other publications. This observation may suggest that the increase of maternal age could be potentially associated with the developing of insulin resistance in GDM patients. However, elderly pregnant women have also a higher BMI index, and it could indicate that adipose tissue may also have an influence on LCN2 levels. But we did not observe a similar correlation in chemerin and apelin analysis in the same group of patients.

A few studies have been published analyzing the role of LCN2 as a predictor of GDM [41, 45]. D'Anna et al. revealed that in women who developed GDM in the previous 12 months, in the first trimester of pregnancy, circulating LCN2 level was markedly higher in patients who subsequently developed GDM. Median serum LCN2 concentrations were positively correlated with HOMA-IR. However, they failed to demonstrate a correlation between LCN2 and pregestational BMI, maternal age, or birth weight [45].

Sweeting et al. in their study tried to find the best risk prediction model for GDM. The authors observed higher LCN2 levels in women who developed GDM. They observed a 10% increase in median MoM LCN2 values in women with GDM and suggested that so small differences, as compared to D'Anna et al. study, presumably reflect the impact of ethnicity on biomarker associations with GDM [41].

In our study, we observed in both groups the positive correlation between chemerin and LCN2 levels. However, the potential physiological and pathological importance of our observations need further explanation. The univariate linear regression model which was performed for chemerin and LCN2 has shown that the growth of each substance serum level similarly increases the likelihood of the GDM incidence in the analyzed group of patients—18% for each 10 ng/ml of chemerin and 20% for each 1 ng/ml of LCN2 (CI 95%, OR: 1.180 vs. 0.200, respectively). In the multiple linear regression analysis of the women with gestational diabetes, we have noticed that the adjusted *R*-square for chemerin was markedly increased as compared to lipocalin (46.1 vs. 20.60, respectively). However, it is important to remember that the main goal of this research was not to describe the cut-off levels of these adipokines in women at 24-28 weeks of gestation when the OGTT is performed.

Gestational diabetes is considered to be an inflammatory disease. Expression of LCN2 in adipose tissue and liver can be induced by lipopolysaccharides, suggesting that LCN2 may be an acute-phase protein. It is suggested that LCN2 may be a significant key to the pathogenesis of inflammation, leading to insulin resistance, followed by an increase in fasting plasma glucose and fasting plasma insulin [42, 46]. However, further studies are needed to evaluate the role of LCN2 in the pathogenesis and prediction of GDM.

In our study, a positive correlation between chemerin and lipocalin 2 levels was observed in both groups. There is a lack of data in the literature regarding the possible explanation of such relationship and clinical importance. We can only hypothesize that our observations can also confirm the possible role of these adipokines in the pathophysiology of gestational diabetes. Thus, the presented results suggest that chemerin serum level evaluation appears to be a more reliable independent predictor of the GDM in future analysis.

The physiological role of apelin is not well known. Animal studies showed that apelin had a glucose-lowering effect correlated with stimulation of glucose utilization in adipose tissue and skeletal muscle from normal and obese insulin-resistant mice [47]. An increased plasma concentration of apelin was noticed in animal models of obesity correlated with hyperinsulinemia. Boucher et al. confirmed that in the obese men and mice, both plasma apelin and insulin values were markedly increased, suggesting that apelin homeostasis is impaired in obesity and indicating that the higher value of plasma insulin could support an increase in blood levels of apelin. Thus, apelin overproduction by adipose tissue may be involved in several obesity-related disturbances [23].

Higher levels of apelin were noticed in patients suffering from T2DM [48]. It has been suggested that apelin secretion can be modulated by proinflammatory adipocytokines, the levels of which are higher in insulin resistance. Daviaud et al. reported a positive correlation between apelin and TNF-*α* expression in adipose tissue and revealed a direct upregulation of apelin expression in both human and mouse adipocytes by TNF-a [49].

There are also some controversies in the literature regarding apelin levels during physiological pregnancy and pregnancy complicated by GDM, and the data are very limited. In the study performed by Kourtis et al., in non-GDM patients, apelin levels were significantly lower in pregnant women than in nonpregnant [50]. In our study, the apelin levels were not statistically significantly higher in the GDM group as compared to the control one. Similar observations have been described by Aslan et al. [51]. However, they measured the apelin levels at the time of delivery. Telejko et al. observed no significant differences in plasma apelin concentrations between the GDM and non-GDM women [52]. The decreased levels of apelin have been revealed by Boyadzhieva et al. and Oncul et al. [53, 54]. In Boyadzhieva et al's study, apelin concentration was significantly lower in the GDM group during the pregnancy. However, there were no statistically significant differences in postpartum groups and no significant correlations between apelin levels and metabolic parameters [53]. In the study conducted by Oncul et al., they also analyzed the maternal and cord blood apelin levels [54].

The cord blood apelin concentrations were significantly lower in GDM women than in the control group. They suggest that GDM appears to modify fetoplacental apelin metabolism, but apelin cannot directly regulate maternal insulin sensitivity [54]. The opposite results of cord blood apelin have also been published [46]. In the study of Aslan et al., the cord blood apelin levels were comparable in the GDM group and in the control one. They also noticed that levels of apelin in the serum of the mothers had a positive correlation with their respective cord blood concentrations. However, in our study, we did not investigate the concentrations of apelin in cord blood, and a full comparison of our results with these observations cannot be performed. Aslan et al. observed the negative association between serum and cord blood apelin values and the gestational age and birth weight [51]. No correlations between serum and cord blood apelin values, maternal age, fasting glucose and insulin levels, BMI, and HOMA-IR were revealed. In our study, we also found no correlations between apelin, clinical, and demographic parameters, but we measured parameters at 24-29 weeks of gestation and, as in chemerin and LCN2 results, we did not analyze the insulin levels and HOMA-IR index.

In our study, there was also no correlation between chemerin and apelin levels and LCN2 and apelin levels. Thus, our results suggest that GDM has no impact on circulating apelin levels.

The differences between our findings and those published in the literature could be explained by the differences in the study protocols and selection process of patient including the week of gestation at blood sampling—the second or third trimester, the type of gestational diabetes—a dietary treatment or insulin treatment which may suggest the severity of metabolic disturbances, the week of pregnancy at the diagnosis of GDM—the first (possible prepregnancy impaired glucose tolerance) or second trimester (“typical” gestational diabetes), the pregestational BMI value or at enrolling to the studies.

The significance of adipokines in the pathogenesis of GDM is still not well known, and none of them have been used as an early predictor for the development of GDM.

Screening for GDM with a 75 g oral glucose tolerance test at 24–28 weeks' gestation and diagnosing GDM in this period of pregnancy have been questioned due to the potential delay in accomplishing the positive effects of pharmacological therapy, diet, and lifestyle modifications. Identifying patient at risk for GDM is essential in the first trimester of pregnancy to minimize maternal and neonatal mortality and morbidity. A limited number of publications have prospectively analyzed the correlation of the HOMA-IR, glycosylated hemoglobin, sex hormone-binding globulin, and cholesterol panel values as a marker for prediction subsequent GDM in low-risk pregnancies during the first trimester of pregnancy but they have low sensitivity and positive predictive value, especially in overweight and obese women. None of these markers have proven adequate to be used in the clinical screening. Mainly, increased HOMA-IR values have been suggested to be associated with GDM. However, the range of HOMA-IR values is wide in women with GDM, and cut-off level is ambiguous [55–57].

## 5. Conclusion

Gestational diabetes mellitus is a widespread condition observed in a large population of pregnant patients. The precise role of adipokines in the pathogenesis of GDM is still not well known. To the best of our knowledge, we did not find in the literature the comparison of circulating levels of chemerin, lipocalin 2, and apelin in the same group of patients with GDM and healthy pregnant women.

We can speculate that these adipokines could potentially be established as novel biomarkers for the early diagnosis of GDM. We hope that our findings will be useful to determine guidelines, in which adipokines may become a novel biomarker in GDM prediction, especially when early pregnancy is concerned. However, further prospective studies are required to evaluate chemerin and lipocalin 2 in the first trimester of pregnancy as a marker of GDM, before the period of pregnancy when the OGTT is performed. It should be remembered that maternal obesity influences the expression of several adipokines in the placenta and in the adipose tissue. Due to these reasons, the correlations between the investigated adipokines and pregnancy-related conditions should be interpreted separately referring to maternal pregestational BMI and pregnancy weight gain, and this problem should be considered in further studies.

## Figures and Tables

**Table 1:** Baseline clinical characteristics (mean and standard deviation; median and range 25-75 percentile for gravidity).

	GDM group (*n* = 153)	Uncomplicated pregnancy group (*n* = 84)	*p* value
Maternal age (years)	27.59 (4.87)	27.23 (4.67)	NS
Gravidity	2 (1-2.5)	2 (1-3)	NS
Pregestational BMI (kg/m^2^)	23.71 (2.64)	22.81 (2.05)	*p* < 0.05
BMI at blood collection (kg/m^2^)	26.63 (2.11)	26.13 (1.71)	NS
EFW at blood collection (g)	920.0 (187.8)	961.2 (168.5)	NS
Weeks of gestation at blood collection	26.54 (1.41)	26.81 (1.26)	NS

GDM: gestational diabetes mellitus; BMI: body mass index; EFW: estimated fetal weight; *p*: statistical significance; NS: statistically not significant.

**Table 2:** Chemerin, lipocalin 2, apelin, and glucose concentrations in both groups (mean and standard deviation).

	GDM group (*n* = 153)	Uncomplicated pregnancy group (*n* = 84)	*p*
Chemerin (ng/mL)	259.55 (63.24)	211.00 (49.38)	*p* < 0.0001
Lipocalin 2 (ng/mL)	40.49 (15.73)	20.63 (7.48)	*p* < 0.0001
Apelin (pg/ml)	10816.45 (7329.52)	9988.24 (5056.90)	*p* = 0.71
Glucose (mmol/L)			
0'	5.20 (0.38)	4.46 (0.39)	*p* < 0.00001
60'	10.06 (1.12)	7.59 (1.41)	*p* < 0.00001
120'	8.71 (1.05)	6.72 (1.19)	*p* < 0.00001

GDM: gestational diabetes mellitus; *p*: statistical significance; NS: statistically not significant.

**Table 3:** Correlation between chemerin, lipocalin, apelin levels, maternal age, gravidity, BMI, EFW, gestational age, and glucose concentrations in OGTT in GDM patients and control group.

	GDM						Control					
	Chemerin		Lipocalin 2		Apelin		Chemerin		Lipocalin 2		Apelin	
	*R*	*p*	*R*	*p*	*R*	*p*	*R*	*p*	*R*	*p*	*R*	*p*
Chemerin			0.631	*p* < 0.0001	0.005	NS			0.635	*p* < 0.0001	-0.048	NS
Lipocalin 2	0.631	*p* < 0.0001			-0.067	NS	0.635	*p* < 0.0001			-0.016	NS
Maternal age (years)	0.157	NS	0.217	*p* < 0.01	-0.108	NS	0.024	NS	0.183	NS	-0.060	NS
Gravidity	0.090	NS	0.130	NS	0.040	NS	0.106	NS	0.216	*p* < 0.05	0.054	NS
Pregestational BMI (kg/m^2^)	0.775	*p* < 0.00001	0.467	*p* < 0.0001	-0.105	NS	0.500	*p* < 0.0001	0.311	*p* < 0.01	0.003	NS
BMI at blood collection (kg/m^2^)	0.693	*p* < 0.00001	0.394	*p* < 0.001	-0.087	NS	0.493	*p* < 0.0001	0.276	*p* < 0.05	0.054	NS
EFW at blood collection (g)	-0.032	NS	-0.063	NS	-0.145	NS	-0.060	NS	0.091	NS	0.055	NS
Weeks of gestation at blood collection	-0.005	NS	-0.066	NS	-0.156	NS	-0.094	NS	0.084	NS	0.005	NS
Glucose												
0'	0.528	*p* < 0.0001	0.266	*p* < 0.01	0.053	NS	0.817	*p* < 0.0001	0.553	*p* < 0.0001	0.074	NS
60'	0.731	*p* < 0.0001	0.425	*p* < 0.001	0.011	NS	0.740	*p* < 0.0001	0.511	*p* < 0.0001	-0.122	NS
120'	0.703	*p* < 0.0001	0.491	*p* < 0.001	-0.051	NS	0.707	*p* < 0.0001	0.423	*p* < 0.001	-0.047	NS

GDM: gestational diabetes mellitus; BMI: body mass index; EFW: estimated fetal weight; *R*: Spearman correlation's coefficient; *p*: statistical significance; NS: statistically not significant.

## Data Availability

The data used to support the findings of this study are included within the article.

## References

[1] American Diabetes Association (2003). Gestational diabetes mellitus. *Diabetes Care*.

[2] Schmidt M. I., Duncan B. B., Reichelt A. J. (2001). Brazilian Gestational Diabetes Study Group. Gestational diabetes mellitus diagnosed with a 2-h 75-g oral glucose tolerance test and adverse pregnancy outcomes. *Diabetes Care*.

[3] Gilmartin A. B. H., Ural S. H., Repke J. T. (2008). Gestational diabetes mellitus. *Reviews in Obstetrics and Gynecology*.

[4] Petry C. J. (2010). Gestational diabetes: risk factors and recent advances in its genetics and treatment. *The British Journal of Nutrition*.

[5] Blüher M. (2012). Clinical relevance of adipokines. *Diabetes and Metabolism Journal*.

[6] Justman N., Shahak G., Gutzeit O. (2020). Lockdown with a price: the impact of the COVID-19 pandemic on prenatal care and perinatal outcomes in a tertiary care center. *The Israel Medical Association Journal*.

[7] Fedullo A. L., Schiattarella A., Morlando M. (2021). Mediterranean diet for the prevention of gestational diabetes in the COVID-19 era: implications of Il-6 in diabesity. *International Journal of Molecular Sciences*.

[8] Miehle K., Stepan H., Fasshauer M. (2012). Leptin, adiponectin and other adipokines in gestational diabetes mellitus and pre-eclampsia. *Clinical Endocrinology*.

[9] Al-Badri M. R., Zantout M. S., Azar S. T. (2015). The role of adipokines in gestational diabetes mellitus. *Therapeutic Advances in Endocrinology and Metabolism*.

[10] Roman A. A., Parlee S. D., Sinal C. J. (2012). Chemerin: a potential endocrine link between obesity and type 2 diabetes. *Endocrine*.

[11] Fatima S. S., Rehman R., Baig M., Khan T. A. (2014). New roles of the multidimensional adipokine: chemerin. *Peptides*.

[12] Svensson H., Oden B., Eden S., Lonn M. (2014). Adiponectin, chemerin, cytokines, and dipeptidyl peptidase 4 are released from human adipose tissue in a depot-dependent manner: an in vitro system including human serum albumin. *BMC Endocrine Disorders*.

[13] Helfer G., Wu Q.-F. (2018). Chemerin: a multifaceted adipokine involved in metabolic disorders. *Journal of Endocrinology*.

[14] Gutaj P., Sibiak R., Jankowski M. (2020). The role of the adipokines in the most common gestational complications. *International Journal of Molecular Sciences*.

[15] Francis E. C., Li M., Hinkle S. N. (2020). Adipokines in early and mid-pregnancy and subsequent risk of gestational diabetes: a longitudinal study in a multiracial cohort. *BMJ Open Diabetes Research & Care*.

[16] Flower D. R. (1996). The lipocalin protein family: structure and function. *The Biochemical Journal*.

[17] Kjeldsen L., Bainton D. F., Sengelov H., Borregaard N. (1994). Identification of neutrophil gelatinase-associated lipocalin as a novel matrix protein of specific granules in human neutrophils. *Blood*.

[18] Yan L., Borregaard N., Kjeldsen L., Moses M. A. (2001). The high molecular weight urinary matrix metalloproteinase (MMP) activity is a complex of gelatinase B/MMP-9 and neutrophil gelatinase-associated lipocalin (NGAL):. *The Journal of Biological Chemistry*.

[19] Lippi G., Meschi T., Nouvenne A., Mattiuzzi C., Borghi L. (2014). Neutrophil gelatinase-associated lipocalin in cancer. *Advances in Clinical Chemistry*.

[20] Rudolph-Owen L. A., Hulboy D. L., Wilson C. L., Mudgett J., Matrisian L. M. (1997). Coordinate expression of matrix metalloproteinase family members in the uterus of normal, matrilysin-deficient, and stromelysin-1-deficient mice. *Endocrinology*.

[21] Wang Y., Lam K. S., Kraegen E. W. (2007). Lipocalin-2 is an inflammatory marker closely associated with obesity, insulin resistance, and hyperglycemia in humans. *Clinical Chemistry*.

[22] Tatemoto K., Hosoya M., Habata Y. (1998). Isolation and characterization of a novel endogenous peptide ligand for the human APJ receptor. *Biochemical and Biophysical Research Communications*.

[23] Boucher J., Masri B., Daviaud D. (2005). Apelin, a newly identified adipokine up-regulated by insulin and obesity. *Endocrinology*.

[24] Berry M., Pirolli T. J., Jayashankar V. (2004). Apelin has in vivo inotropic effects on normal and failing hearts. *Circulation*.

[25] Hashimoto T., Kihara M., Imai N. (2007). Requirement of apelin-apelin receptor system for oxidative stress-linked atherosclerosis. *The American Journal of Pathology*.

[26] Maury E., Brichard S. M. (2010). Adipokine dysregulation, adipose tissue inflammation and metabolic syndrome. *Molecular and Cellular Endocrinology*.

[27] Briana D. D., Malamitsi-Puchner A. (2009). Reviews: adipocytokines in normal and complicated pregnancies. *Reproductive Sciences*.

[28] World Health Organization (2014). Diagnostic criteria and classification of hyperglycaemia first detected in pregnancy. *Diabetes Research and Clinical Practice*.

[29] Kasher-Merona M., Mazaki-Tovi S., Barhod E. (2014). Chemerin concentrations in maternal and fetal compartments: implications for metabolic adaptations to normal human pregnancy. *Journal of Perinatal Medicine*.

[30] Li X.-M., Ji H., Li C.-J. (2015). Expression de la chemerine chez la femme enceinte chinoise atteinte ou non de diabete gestationnel. *Annales d'endocrinologie*.

[31] Yang X., Quan X., Lan Y. (2017). Serum chemerin level during the first trimester of pregnancy and the risk of gestational diabetes mellitus. *Gynecological Endocrinology*.

[32] Ademoglu E., Berberoglu Z., Dellal F. D. (2015). Higher levels of circulating chemerin in obese women with gestational diabetes mellitus. *Acta Endocrinologica*.

[33] Fatima S. S., Alam F., Chaudhry B., Khan T. A. (2017). Elevated levels of chemerin, leptin, and interleukin-18 in gestational diabetes mellitus. *The Journal of Maternal-Fetal & Neonatal Medicine*.

[34] Lehrke M., Becker A., Greif M. (2009). Chemerin is associated with markers of inflammation and components of the metabolic syndrome but does not predict coronary atherosclerosis. *European Journal of Endocrinology*.

[35] van Poppel M. N. M., Zeck W., Ulrich D. (2014). Cord blood chemerin: differential effects of gestational diabetes mellitus and maternal obesity. *Clinical Endocrinology*.

[36] Barker G., Lim R., Rice G. E., Lappas M. (2012). Increased chemerin concentrations in fetuses of obese mothers and correlation with maternal insulin sensitivity. *The Journal of Maternal-Fetal & Neonatal Medicine*.

[37] Zhou Z., Chen H., Ju X., Sun M. (2018). Circulating chemerin levels and gestational diabetes mellitus: a systematic review and meta-analysis. *Lipids in Health and Disease*.

[38] Zhang J., Chi H., Xiao H. (2017). Interleukin 6 (IL-6) and tumor necrosis factor *α* (TNF-*α*) single nucleotide polymorphisms (SNPs), inflammation and metabolism in gestational diabetes mellitus in Inner Mongolia. *Medical Science Monitor*.

[39] Arikan D. C., Ozkaya M., Adali E. (2011). Plasma lipocalin-2 levels in pregnant women with pre-eclampsia, and their relation with severity of disease. *The Journal of Maternal-Fetal & Neonatal Medicine*.

[40] Cakal E., Ozkaya M., Engin-Ustun Y., Ustun Y. (2011). Serum lipocalin-2 as an insulin resistance marker in patients with polycystic ovary syndrome. *Journal of Endocrinological Investigation*.

[41] Sweeting A. N., Wong J., Appelblom H. (2019). A novel early pregnancy risk prediction model for gestational diabetes mellitus. *Fetal Diagnosis and Therapy*.

[42] Lou Y., Wu C., Wu M., Xie C., Ren L. (2014). The changes of neutrophil gelatinase-associated lipocalin in plasma and its expression in adipose tissue in pregnant women with gestational diabetes. *Diabetes Research and Clinical Practice*.

[43] Edelstam G., Lowbeer C., Kral G., Gustafsson S. A., Venge P. (2001). New reference values for routine blood samples and human neutrophilic lipocalin during third-trimester pregnancy. *Scandinavian Journal of Clinical and Laboratory Investigation*.

[44] Kocot J., Dziemidok P., Kiełczykowska M., Kurzepa J., Szcześniak G., Musik I. (2018). Is there any relationship between plasma 25-hydroxyvitamin D3, adipokine profiles and excessive body weight in type 2 diabetic patients?. *International Journal of Environmental Research and Public Health*.

[45] D’Anna R., Baviera G., Corrado F., Giordano D., Recupero S., Di Benedetto A. (2009). First trimester serum neutrophil gelatinase associated lipocalin in gestational diabetes. *Diabetic Medicine*.

[46] Zhang J., Wu Y., Zhang Y., Leroith D., Bernlohr D. A., Chen X. (2008). The role of lipocalin 2 in the regulation of inflammation in adipocytes and macrophages. *Molecular Endocrinology*.

[47] Dray C., Knauf C., Daviaud D. (2008). Apelin stimulates glucose utilization in normal and obese insulin-resistant mice. *Cell Metabolism*.

[48] Castan-Laurell I., Dray C., Attane C., Duparc T., Knauf C., Valet P. (2011). Apelin, diabetes, and obesity. *Endocrine*.

[49] Daviaud D., Boucher J., Gesta S. (2006). TNF*α* up-regulates apelin expression in human and mouse adipose tissue. *The FASEB Journal*.

[50] Kourtis A., Gkiomisi A., Mouzaki M. (2011). Apelin levels in normal pregnancy. *Clinical Endocrinology*.

[51] Aslan M., Celik O., Celik N. (2012). Cord blood nesfatin-1 and apelin-36 levels in gestational diabetes mellitus. *Endocrine*.

[52] Telejko B., Kuzmicki M., Wawrusiewicz-Kurylonek N. (2010). Plasma apelin levels and apelin/APJ mRNA expression in patients with gestational diabetes mellitus. *Diabetes Research and Clinical Practice*.

[53] Boyadzhieva M., Atanasova I., Zacharieva S., Kedikova S. (2013). Adipocytokines during pregnancy and postpartum in women with gestational diabetes and healthy controls. *Journal of Endocrinological Investigation*.

[54] Oncul M., Tuten A., Erman H., Gelisgen R., Benian A., Uzun H. (2013). Maternal and cord blood apelin, resistin and visfatin levels in gestational diabetes mellitus. *Minerva Medica*.

[55] Smirnakis K. V., Plati A., Wolf M., Thadhani R., Ecker J. L. (2007). Predicting gestational diabetes: choosing the optimal early serum marker. *American Journal of Obstetrics and Gynecology*.

[56] Ozcimen E. E., Uckuyu A., Ciftci F. C., Yanik F. F., Bakar C. (2008). Diagnosis of gestational diabetes mellitus by use of the homeostasis model assessment-insulin resistance index in the first trimester. *Gynecological Endocrinology*.

[57] Kumru P., Arisoy A., Erdogdu E. (2016). Prediction of gestational diabetes mellitus at first trimester in low-risk pregnancies. *TJOG*.

